# Silymarin conserves the efficacy of quinolone and sulfonamide in Nile tilapia *(Oreochromis niloticus)* subjected to aflatoxicosis and *Streptococcus agalactiae* infection

**DOI:** 10.1038/s41598-026-54047-5

**Published:** 2026-06-03

**Authors:** Nourhan A. Haggag, Mohamed Elbadawy, Ashraf ElKomy, Ahmed H. Sherif

**Affiliations:** 1 Pharmacology Department, Agriculture Research Center ARC, Animal Health Research Institute AHRI, Tanta, 12619 Egypt; 2 Department of Pharmacology, Faculty of Veterinary Medicine, Banha University, Banha, 13736 Egypt; 3 Fish Diseases Department, Agriculture Research Center ARC, Animal Health Research Institute AHRI, Kafrelsheikh, 12619 Egypt

**Keywords:** *Silybum marianum*, Cytokine, Antioxidant, *Oreochromis niloticus*, Aflatoxicosis, Biochemistry, Immunology, Microbiology

## Abstract

Aflatoxins (AFB) are adventitious plant toxins that act as immunosuppressants. Experimental Nile tilapia were divided into eight groups: AFB0 (control, uncontaminated diet), AFB50 (50 ppbAFB_1_), AFB100 (100 ppbAFB_1_), AFB150 (150 ppbAFB_1_), AFB0 + Sil (0 ppbAFB_1_+Sil), AFB50 + Sil (50 ppbAFB_1_+Sil), AFB100 + Sil (100 ppbAFB_1_+Sil), and AFB150 + Sil (150 ppbAFB_1_+Sil). The AFB_1_-residues in fish liver (AFB0, AFB50, AFB100, and AFB150) were 0.34, 0.54, and 0.82 ppb, respectively, while traces were recorded in muscles. Sil-supplemented fish had lower residues than other contaminated groups, which were still higher than AFB0. The gene expression of *GPx*,* SOD*, *IL-1β*, and *TNF-α* was significantly and linearly decreased with increasing aflatoxin levels, whereas Sil-supplementation (AFB50 + Sil) resulted in a high and significant increase of 3.86, 5.37, and 7.85 fold-change, respectively, compared with the fish groups and the control. While, CAT expression increased in response to a low aflatoxin level (AFB50; 2.17-fold-change), it decreased linearly with increasing aflatoxin level. Moreover, *IL-10* expression was significantly higher in fish of AFB100 and AFB150, with 8.7- and 8.85-fold changes, respectively, compared to the control. Significant declines in immunoglobulin *(Ig)M-2* and insulin-like growth factor *(IGF)* gene expression were observed in fish receiving AFB_1_-contaminated diets and Sil could enhance the expression. Innate immunity of the experimental fish was assessed by measuring the following parameters; serum antibacterial activity (SAB), oxidative burst activity (OBA), phagocytic activity (PA), phagocytic index (PI), and lysozyme (LYZ) as well as by challenge with *Streptococcus agalactiae*. Nile tilapia fed AFB_1_-contaminated diets showed immune-antioxidant suppressive status, indicating compromised health and a lowered survival rate (SR%) during bacterial challenge and antibiotic treatment Sil-supplementation could improve SR%.

## Introduction

In the last decade, the consumption of aquatic animal has increased significantly as human food and/or fishmeal production^1^. This has led to raise the demand and consequently prices of fishmeal to produce animal feed, nutritionists have started to recommend partially or completely replacing fishmeal with plant-origin ingredients, which contain many adventitious toxins^2^. Many regulations have set permissible limits for aflatoxin (AFB_1_) levels, which are secreted by *Aspergillus parasiticus*, in livestock feeds at 20 ppb^3^, corn, cereals and cereal-based products in the United States while 10 ppb in the Europe union^4^. Farming Nile tilapia (*Oreochromis niloticus*) is the most prevalent practice of aquaculture in tropical and subtropical regions which characterized by favorable conditions for fungal propagation, such as a humid atmosphere and ambient temperature resulting in AFB_1_ contamination in fish rations. Fishes fed AFB_1_-contaminated feed exhibited a downgrade in physiological status, even at low AFB_1_ levels^5^. The sensitivity to AFB_1_ differed according to fish species however the main sites of accumulation are tissues of liver, kidney, and muscles^6,7^, AFB_1_ more than 350 ppb in the feed resulted in remarked accumulation in liver and muscles of jundia´ (*Rhamdia quelen*) exposed to^8^, residues detected in hepatic and muscular tissues after 90 days of exposure to 50 ppb AFB_1_. In contrast, after 120 days muscular residues was equal the diet level^9^.

Fish pathologists have sought to minimize the use of antimicrobials in food-fish treatment, so in the last decades herbal medicine has attracted their attention mainly for its antioxidant-immune properties^10–12^. In this work, AFB1 adversely impacted liver health thereby decreasing antibiotic metabolism resulting in a decrease in their efficacy. Silymarin (Sil) is an antioxidant patent pharmaceutical, extracted from *Silybum marianum* seeds, containing high levels of polyunsaturated essential fatty acids, vitamin E, and sterols^13^. Anti-inflammatory properties of Sil ameliorate liver damage, mitigate oxidative stress, and improve the immune responses in rainbow trout^14–16^. Generally, the hepatoprotective effect of Sil can be manifested by AST and ALT decrease in serum in stressed fish^17,18^.

Antibiotic administration, usually via medicated feed, is a very common practice, especially in fish farms facing infectious disease outbreaks, Ciprofloxacin (Cip) a second-generation fluoroquinolone, and sulfonamides are the most prescribed antibiotics in aquatic animals treatment^19–21^. Also, sulfonamides (SAs) are usually used in combination with a pyrimidine potentiator (potentiated sulfa drugs), in a ratio of 5:1, for example, sulfadiazine (SDZ) and trimethoprim (TMP), to broaden the spectrum of bactericidal activity^22,23^, including *Vibrio alginolyticus*,* V. ordali*,* Listonella (Vibrio) anguillarum*, and *Photobacterium damselae spp. Piscicida*^24^.

In this study, the impacts of aflatoxicosis on Nile tilapia immunity and vulnerability to *S. agalactiae* are examined. Antioxidants *GPx* and *SOD*, cytokines *IL-1β* and *TNF-α*, and innate immunity were assessed by RT-PCR, as well as by measuring phagocytic cell activity and serum antibacterial activity. Finally, disease resistance was assessed by conducting an in vivo bacterial challenge with *Streptococcus agalactiae.*

## Materials and methods

### Experimental design and accommodation

Two hundred and forty Nile tilapia (*Oreochromis niloticus*) were obtained and transported from local freshwater fish farms (um-sin village, Kafrelshiekh, Egypt) to the experimental wet laboratory, Animal Health Research Institute AHRI. At the arrival, fish were disinfected with a 10 ppm iodine bath for 10 s (Betadine^®^, 5% of povidone-iodine, Nile Company for Pharmaceuticals). Transported fish were stocked in one fiber-glass tank (3 × 1.5 × 1 m) containing dechlorinated and clean tap water for two weeks under observation. After two-week acclimatization, fish weighing 50.8 ± 0.2 g were tranquillized using 40 mg/L tricaine methanesulfonate (MS-222, Syndel, Canada) and allocated into twenty-four glass aquaria (50 × 50 × 40 cm), 10 fish/aquarium, 80-liter water content^25,26^.

Fish fed aflatoxin (AFB_1_)-contaminated diets (0, 50, 100, 150 ppb)^27,28^, and dietary silymarin (Sil) was used as a feed additive at a dose of 100 mg/kg fish feed. Eight experimental groups in triplicate according to the levels of AFB1 and Sil; AFB0 (control, uncontaminated diet), AFB50 (50 ppb), AFB100 (100 ppb), AFB150 (150 ppb), AFB0 + Sil (0 ppb + Sil), AFB50 + Sil (50 ppb + Sil), AFB100 + Sil (100ppb + Sil), and AFB150 + Sil (150 ppb + Sil). The feeding trial lasted for 84 days. Commercial fish food (in the form of pellets) was soaked in water. It was blended into a paste, and AFB1 plus silymarin were added at the determined doses. Gelatin (Nutri-B-Gel) produced by Canal Aqua Cure (Port-Said, Egypt) was then mixed with the formed paste to a final level of 5% w/w to enhance the consistency and viscoelastic properties of the food. The diet pastes were syringed into 2 mm syringes. Then the diets were dried and similarly cut into uniform pellets. Fish were fed a 3% their body weight diet, which was offered in two halves per day at 09.00 am and 03.00 pm. The amount of feed was adjusted based on the weekly-calculated fish weight.

- Silymarin (Sil) was produced by Safe for pharmaceutical products, new Borg El-Arab, Egypt, in pharmaceutical form of capsules containing 140 mg (Batch No.: 0920468/2020).
- Feed 10 mg of ciprofloxacin/kg of body weight/day for 10 days three days post-infection, trade name ciprofar (500 mg). Pharco pharmaceuticals (Reg. No.: 21515/2012). Amirya-Alexandria, Egypt^29^. Feed 50 mg/kg of b.w. sulfadiazine (SDZ) and trimethoprim (TMP) (200: 40 mg) for 10 days three days post-infection, trade name colitrim (500 mg). Pharco pharmaceuticals (Reg. No.: 1544/2010, Batch No.: L231146) Pharma swede, Egypt^29^.
- Aflatoxin (AFB_1_) was prepared according to the method provided by Abdelhamid and Mahmoud^30^, *Aspergillus parasiticus (*NRRL 2999) was grown on corn pellets. Harvested spore suspension was inoculated into mycotic media which consisted of yeast extract-sucrose broth containing 2% yeast extract and 20% sucrose, and incubated for 9 days at 25–29 °C. The concentration of formed AFB_1_ was assessed using quantitative thin-layer chromatography TLC^31^.

### Determination of genes expression of immune-antioxidant

To obtain hepatic tissues, experimental fish were killed using a MS-222 bath (250 mg/L) for 10 min^25^. The impacts of Sil on immune-antioxidant markers of Nile tilapia exposed to aflatoxicosis were evaluated. The expression of immune-antioxidant-related genes glutathione peroxidase (*GPx)*, catalase *(CAT)*, superoxide dismutase *(SOD)*, interleukin *(IL)−1β*, tumour necrosis factor *(TNF-α)*, *IL-10*, immunoglobulin *(Ig)M-2*, and Insulin-like growth factor *(IGF)1* was determined. Firstly, the RNA was extracted from hepatic tissues using TRizol reagent (iNtRON Biotechnology Inc., Korea), and then assessed for its quality and quantity then preserved at − 80 °C until further procedures. The expression titer was calculated using quantitative real-time polymerase chain reaction (RT-qPCR), while *β-actin* was used as a housekeeping gene and for sample normalization, as it is ubiquitously expressed and highly stable across various cell types^32^ (Table 1). The results were illustrated using Eq. 2^−ΔΔ^CT^33^.

**Table 1:** Primer list used in the study, annealing temperature (Ta), base pair (bp) of primers used in Real Time PCR (RT-PCR).

Gene	Strand (5’−3’)	Sequence	Accession number
*GPx*	F	CCAAGAGAACTGCAAGAACGA	NM_001279711.1
	R	CAGGACACGTCATTCCTACAC	
*CAT*	F	TCCTGAATGAGGAGGAGCGA	JF801726.1
	R	ATCTTAGATGAGGCGGTGATG	
*SOD*	F	GGTGCCCTGGAGCCCTA	JF801727.1
	R	ATGCGAAGTCTTCCACTGTC	
*IL-1β*	F	TGCTGAGCACAGAATTCCAG	XM_019365841.2
	R	GCTGTGGAGAAGAACCAAGC	
*TNF-α*	F	CCAGAAGCACTAAAGGCGAAGA	AY428948.1
	R	CCTTGGCTTTGCTGCTGATC	
*IL-10*	F	CTGCTAGATCAGTCCGTCGAA	XM_013269189.3
	R	GCAGAACCGTGTCCAGGTAA	
*IgM-2*	F	CCACTTCAACTGCACCCACT	KC677037.1
	R	TGGTCCACGAGAAAGTCACC	
*IGF1*	F	GTTTGTCTGTGGAGAGCGAGG	NM_001279503.1
	R	GAAGCAGCACTCGTCCACG	
*β-actin*	F	GCATCACACCTTCTACAACGA	AA566386
	R	TGGCGGGGGTGTTGAAGGTCT	

*GPx* glutathione peroxidase, *SOD* superoxide dismutase, *CAT* catalase, *IL-1β* Interleukin-1 beta, *TNF- α* tumour necrosis factor alpha, *IgM-2* immunoglobulin M-2, *IGF1* Insulin like growth factor 1.

### Innate immunity

#### Oxidative burst activity (OBA)

The activity of experimental fish heterophils was assessed by measuring the glass adherent ability (nitroblue tetrazolium test) using the method developed by Anderson et al.^34^. One drop of heparinized blood sample of Nile tilapia (three fish from each group) was placed onto a glass cover slip then incubated at room temperature (25 ℃) for 30 min, cover slips were carefully and gently washed under running tap water then dried and stained with 0.2% filtered NBT solution (Fluka Buchs, Co. Switzerland), under a light microscope, dark-blue cells were positive counted as active heterophils.

#### Phagocytosis assay

Phagocytic activity (PA) and index (PI) of experimental fish leukocytes were determined using the methods of Faulmann et al.^35^, in which immune cells were isolated, mixed with *C. albicans*, and incubated for one hour at 27 °C (CO_2_ 5%)^36^. The assessment was done following the equations below:

$$\:\mathrm{P}\mathrm{A}\left(\mathrm{\%}\right)\:=\frac{\mathrm{N}\mathrm{o}.\:\mathrm{o}\mathrm{f}\:\mathrm{i}\mathrm{n}\mathrm{g}\mathrm{e}\mathrm{s}\mathrm{t}\mathrm{i}\mathrm{n}\mathrm{g}\:\mathrm{p}\mathrm{h}\mathrm{a}\mathrm{g}\mathrm{o}\mathrm{c}\mathrm{y}\mathrm{t}\mathrm{e}\mathrm{s}\:}{\mathrm{t}\mathrm{o}\mathrm{t}\mathrm{a}\mathrm{l}\:\mathrm{N}\mathrm{o}.\:\mathrm{o}\mathrm{f}\:\mathrm{p}\mathrm{h}\mathrm{a}\mathrm{g}\mathrm{o}\mathrm{c}\mathrm{y}\mathrm{t}\mathrm{e}\mathrm{s}}\times\:\:100$$

$$\:\mathrm{P}\mathrm{I}\left(\mathrm{c}\mathrm{e}\mathrm{l}\mathrm{l}\right)\:=\frac{\mathrm{N}\mathrm{o}.\:\mathrm{o}\mathrm{f}\:\mathrm{i}\mathrm{n}\mathrm{g}\mathrm{e}\mathrm{s}\mathrm{t}\mathrm{e}\mathrm{d}\:C.\:albicans\:\mathrm{c}\mathrm{e}\mathrm{l}\mathrm{l}\mathrm{s}\:\:}{\mathrm{N}\mathrm{o}.\:\mathrm{o}\mathrm{f}\:\mathrm{i}\mathrm{n}\mathrm{g}\mathrm{e}\mathrm{s}\mathrm{t}\mathrm{i}\mathrm{n}\mathrm{g}\:\mathrm{p}\mathrm{h}\mathrm{a}\mathrm{g}\mathrm{o}\mathrm{c}\mathrm{y}\mathrm{t}\mathrm{e}\mathrm{s}}$$

#### Serum antibacterial activity (SAA)^37^

Blood samples were collected without an anticoagulant to obtain experimental Nile tilapia sera. Equal volumes (100 µl) of sera and *Aeromonas hydrophila* suspensions (2 × 10^8^ CFU) were mixed and incubated at room temperature (25 °C) for one hour, then the mixtures of sera. The bacterial were incubated on blood agar at 27 °C for 24 h, and the bacteria colonies were counted and presented as colony forming unit (CFU).

#### Lysozyme activity^38^

The activity was determined in Nile tilapia serum using Micrococcus luteus as a substrate in the turbidimetric method. The obtained results indicated the quantity of enzyme activity, recorded as 0.001/min in 1mL of fish serum.

### Challenge test, mortality rate, re-isolation and relative level of protection

The vulnerability of experimental Nile tilapia to bacterial infection was assessed by conducting an experimental infection with *Streptococccus agalactiae*, a bacterial strain previously isolated, identified, and submitted to the NCBI database under accession number OL471408. After the feeding trial, Nile tilapia (10 fish) from each group were randomly picked and intraperitoneally (IP) injected with at a dose of 10% LD_50_ (3 × 10^5^ cfu) of *S. agalactiae*^39^, LD was determined using^40^ method, negative controls were ten of control fish which were injected with normal saline solution at a concentration of 0.65%^41^. The injected Nile tilapia were observed for two weeks. The mortality rates (MR %) and relative protection levels (RLP)^42^ for Sil, Cip, and SDZ&TMP were calculated using the equations below.

$$\:\mathrm{M}\mathrm{R}\mathrm{\%}=\frac{\mathrm{n}\mathrm{u}\mathrm{m}\mathrm{b}\mathrm{e}\mathrm{r}\:\mathrm{o}\mathrm{f}\:\mathrm{d}\mathrm{e}\mathrm{a}\mathrm{t}\mathrm{h}\mathrm{s}\:\mathrm{i}\mathrm{n}\:\mathrm{a}\:\mathrm{s}\mathrm{p}\mathrm{e}\mathrm{c}\mathrm{i}\mathrm{f}\mathrm{i}\mathrm{c}\:\mathrm{p}\mathrm{e}\mathrm{r}\mathrm{i}\mathrm{o}\mathrm{d}}{\mathrm{t}\mathrm{o}\mathrm{t}\mathrm{a}\mathrm{l}\:\mathrm{p}\mathrm{o}\mathrm{p}\mathrm{u}\mathrm{l}\mathrm{a}\mathrm{t}\mathrm{i}\mathrm{o}\mathrm{n}\:\mathrm{d}\mathrm{u}\mathrm{r}\mathrm{i}\mathrm{n}\mathrm{g}\:\mathrm{t}\mathrm{h}\mathrm{a}\mathrm{t}\:\mathrm{p}\mathrm{e}\mathrm{r}\mathrm{i}\mathrm{o}\mathrm{d}}\times\:\:100$$

$$\:\mathrm{R}\mathrm{L}\mathrm{P}\mathrm{\%}=(1-\frac{\mathrm{\%}\mathrm{d}\mathrm{e}\mathrm{a}\mathrm{t}\mathrm{h}\mathrm{s}\:\mathrm{i}\mathrm{n}\:\mathrm{t}\mathrm{h}\mathrm{e}\:\mathrm{t}\mathrm{r}\mathrm{e}\mathrm{a}\mathrm{t}\mathrm{e}\mathrm{d}\:\mathrm{g}\mathrm{r}\mathrm{o}\mathrm{u}\mathrm{p}}{\mathrm{\%}\mathrm{d}\mathrm{e}\mathrm{a}\mathrm{t}\mathrm{h}\mathrm{s}\:\mathrm{i}\mathrm{n}\:\mathrm{t}\mathrm{h}\mathrm{e}\:\mathrm{c}\mathrm{o}\mathrm{n}\mathrm{t}\mathrm{r}\mathrm{o}\mathrm{l}\:\mathrm{g}\mathrm{r}\mathrm{o}\mathrm{u}\mathrm{p}})\times\:\:100$$

Treatment with SDZ & TMP and Cip started after three days of the onset of the bacterial infection.

### Statistical analyses

The effects of AFB1 on Nile tilapia health and antioxidant-immune status, as well as those of Sil, Cip, and SDZ&TMP, were evaluated by comparing calculated means and standard errors. All values were tested for normality and homogeneity. Groups were compared using ANOVA in SPSS software version 22. P-values for the collected data: if less than 0.05, they were statistically significant using Duncan’s Multiple Range.

## Results

### Analyses of aflatoxins residues

Fish diets containing 50, 100, and 150 ppb of AFB1 were fed to experimental Nile tilapia for 84 days. Analysis showed that AFB1 was 64.2, 122.4, and 171.8 ppb in the formulated diets, respectively. Fish fed aflatoxin-contaminated diets showed graded liver residues of about 0.34, 0.54, and 0.82 ppb, respectively, while traces were recorded in muscle tissues.

### Antioxidant gene expression

**Fig. 1:**
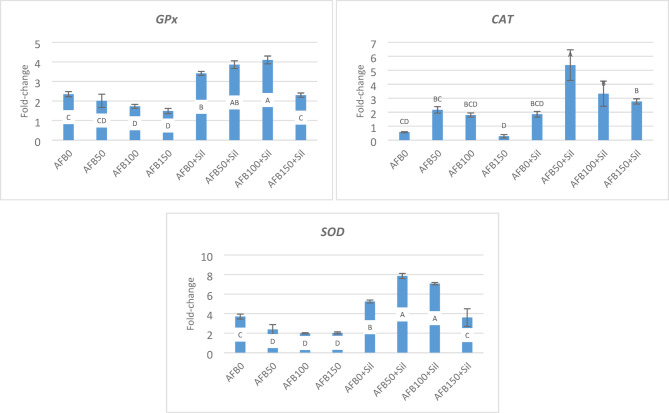
Gene expression of antioxidants in hepatic tissues of the experimental Nile tilapia. Fold-change equal 1.0 by convention for 2^−ΔΔCT^. Different letters in the same column indicate significant differences between values at *P* ≤ 0.05. AFB0 (control, uncontaminated diet), AFB50 (50 ppb AFB_1_), AFB100 (100 ppb AFB_1_), AFB150 (150 ppb AFB_1_), AFB0 + Sil (0 ppb AFB_1_+100 mg silymarin), AFB50 + Sil (50 ppb AFB_1_+100 mg silymarin), AFB100 + Sil (100ppb AFB1 + 100 mg silymarin), and AFB150 + Sil (150 ppb AFB_1_+100 mg silymarin).

In Fig. 1, AFB1 adversely impacted the antioxidant status of the experimental fish after 84 days of exposure, as gene expression of GPx and SOD was significantly and linearly decreased with increasing AFB1 level. In contrast, CAT expression was increased in response to low AFB1 level (AFB50), with a 2.17-fold change, then decreased linearly with increasing AFB1 level, compared to the control, with a 0.57-fold change.

Sil-supplementation resulted in a significant increase in GPx, CAT, and SOD, and the greatest fold change was observed in fish AFB50 + Sil (3.86, 5.37, and 7.85, respectively). Then AFB100 + Sil and AFB150 + Sil had lower values; however, these values were higher than AFB0 (Fig. 1).

### Cytokines, immunoglobulin *(IgM-2)*, and insulin like growth factor1 *(IGF1)* gene expression

**Fig. 2:**
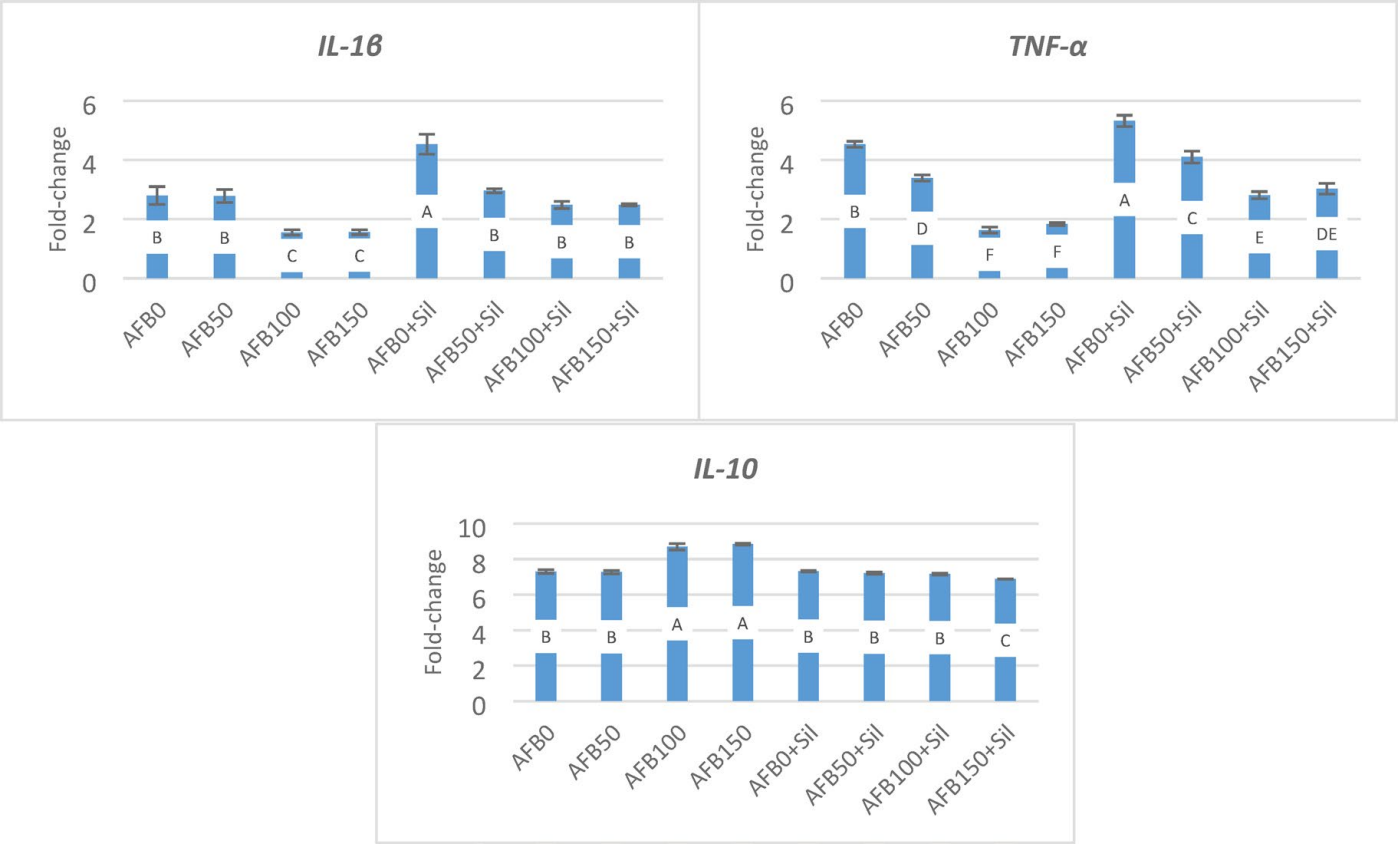
Gene expression of cytokines. Fold-change equal 1.0 by convention for 2^−ΔΔCT^. Different letters in the same column indicate significant differences between values at *P* ≤ 0.05. AFB0 (control, uncontaminated diet), AFB50 (50 ppb AFB_1_), AFB100 (100 ppb AFB_1_), AFB150 (150 ppb AFB_1_), AFB0 + Sil (0 ppb AFB_1_+100 mg silymarin), AFB50 + Sil (50 ppb AFB_1_+100 mg silymarin), AFB100 + Sil (100ppb AFB1 + 100 mg silymarin), and AFB150 + Sil (150 ppb AFB_1_+100 mg silymarin).

In Fig. 2, proinflammatory *IL-1β* and *TNF-α* gene expression (Fig. 2) was linearly decreased in hepatic tissues of fish receiving aflatoxin AFB50, AFB100, and AFB150. While Sil-supplementation (AFB_1_+Sil) restored higher gene expression levels compared to the control AFB0, with 2.8- and 4.53-fold-change, respectively. Whereas, an increase of AFB_1_ levels (AFB50 + Sil, AFB100 + Sil, and AFB150 + Sil) resulted in a linear decline of gene expression values however these values were higher than those received contaminated-diets. While fish (AFB50) had anti-inflammatory *(IL-10)* gene expression (Fig. 2) in the hepatic tissue that was insignificantly different from the control AFB0 (7.3-fold-change), fish in AFB100 and AFB150 were significantly higher, 8.7- and 8.85-fold-change, respectively. Meanwhile, Sil supplementation could counteract aflatoxin’s adverse effects.

**Fig. 3:**
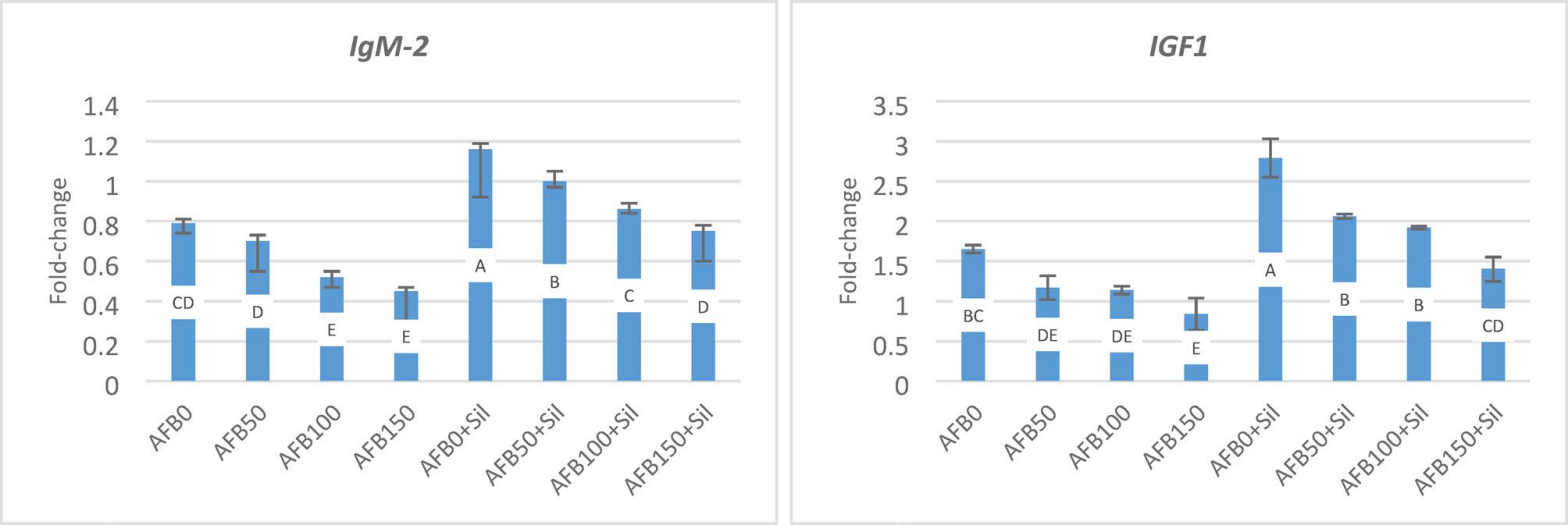
Gene expression of immunoglobulin (IgM-2), and insulin like growth factor (IGF). Fold-change equal 1.0 by convention for 2^−ΔΔCT^. Different letters in the same column indicate significant differences between values at *P* ≤ 0.05. AFB0 (control, uncontaminated diet), AFB50 (50 ppb AFB_1_), AFB100 (100 ppb AFB_1_), AFB150 (150 ppb AFB_1_), AFB0 + Sil (0 ppb AFB_1_+100 mg silymarin), AFB50 + Sil (50 ppb AFB_1_+100 mg silymarin), AFB100 + Sil (100ppb AFB1 + 100 mg silymarin), and AFB150 + Sil (150 ppb AFB_1_+100 mg silymarin).

While dietary AFB_1_ resulted in a significant decline in *IgM-2* gene expression in hepatic tissues (Fig. 3), Sil supplementation could alleviate this withdrawal, meanwhile all alterations were linearly correlated with AFB_1_ levels. Gene expression of IGF was significantly and linearly decreased in all experimental groups following the rise in AFB1 level. AFB0 + Sil was significantly higher (2.70 fold-change) compared to the control (AFB0, 1.65 fold-change), whereas AFB100 + Sil was 1.4 fold-change, which was insignificantly lower than the control (AFB0) (Fig. 2).

Innate immunity of the experimental fish was assessed by measuring the following items: SAB, OBA, PA, PI, and LYZ (Table 2).

Serum of fish fed aflatoxin-contaminated diets showed significant activity (SAB) against A. hydrophila. Activity SAB was negatively associated with AFB1 levels. Compared with AFB0 (control), AFB50 was not significantly different, whereas AFB100 and AFB150 were significantly decreased by 45%, 41.5%, 35.75%, and 29.6%, respectively. Sil-supplementation restored SAB activity; however, at high AFB1 levels, AFB150 + Sil failed to restore SAB activity. Even OBA showed the same trend as SAB; Sil-supplementation did not counteract AFB100 + Sil or AFB150 + Sil, which remained significantly lower than AFB0 (control).

Phagocytosis of immune cells of Nile tilapia was drastically decreased after receiving an aflatoxin-contaminated diet. PA was significantly downregulated in AFB100 and AFB150 compared to AFB0 (control): 16.67% and 3.33%, respectively, and 46.67% in AFB150. However, Sil-supplementation significantly stimulated PA to 53.3% (AFB0 + Sil) and 36.67% (AFB50 + Sil); it did not stimulate those who received high levels of aflatoxin (AFB100 and AFB150). While PI showed insignificant differences between fish in the experimental groups.

In Table 2, he activity of serum LYZ followed the trend of OBA and PA, as Sil supplementation did not restore activity in those with diets contaminated with high AFB1 levels (AFB100 and AFB150).

**Table 2:** Innate immunity parameters in the experimental Nile tilapia.

Items	SAB (%)	OBA (cell-number)	PA (%)	PI (yeast-cell)	LYZ (µg/mL)
AFB0	45^**B**^ ± 0.87	7.33^**AB**^ ± 0.3	46.67^**AB**^ ± 3.3	1.67^**B**^ ± 0.3	0.25^**AB**^ ± 0.06
AFB50	41.60^**BC**^ ± 0.4	6.33^**B**^ ± 0.3	35^**B**^ ± 2.89	1.67^**B**^ ± 0.67	0.13^**CD**^ ± 0.015
AFB100	35.75^**D**^ ± 2.1	4.33^**CD**^ ± 0.67	16.67^**DE**^ ± 8.8	1^**B**^ ± 0.58	0.08^**D**^ ± 0.00
AFB150	29.6^**E**^ ± 0.88	3^**D**^ ± 0.58	3.33^**E**^ ± 3.3	0.33^**B**^ ± 0.3	0.08^**D**^ ± 0.00
AFB0 + Sil	54.2^**A**^ ± 0.5	8.67^**A**^ ± 0.3	53.3^**A**^ ± 6.67	3.3^**A**^ ± 0.3	0.30^**A**^ ± 0.01
AFB50 + Sil	42.30^**BC**^ ± 0.52	6.67^**B**^ ± 0.3	36.67^**AB**^ ± 3.3	1.67^**B**^ ± 0.3	0.20^**BC**^ ± 0.01
AFB100 + Sil	40.47^**C**^ ± 0.26	4.67^**C**^ ± 0.3	33.30^**BC**^ ± 1.67	1^**B**^ ± 0.0	0.15^**CD**^ ± 0.012
AFB150 + Sil	37.1^**D**^ ± 1.68	3.67^**CD**^ ± 0.67	16.67^**DE**^ ± 8.8	0.67^**B**^ ± 0.3	0.09^**D**^ ± 0.005

Different letters in the same column indicate significant differences between values at *P* ≤ 0.05. AFB0 (control, uncontaminated diet), AFB50 (50 ppb AFB_1_), AFB100 (100 ppb AFB_1_), AFB150 (150 ppb AFB_1_), AFB0 + Sil (0 ppb AFB_1_+100 mg silymarin), AFB50 + Sil (50 ppb AFB_1_+100 mg silymarin), AFB100 + Sil (100ppb AFB1 + 100 mg silymarin), and AFB150 + Sil (150 ppb AFB_1_+100 mg silymarin).

### Challenge of experimental fish with *S. agalactiae*

In Table 3, experimental Nile tilapia were challenged with a sub-lethal dose of S. agalactiae (10% of LD50) and treated with SDZ & TMP, and Cip after 3 days from the onset of infection. It was observed that Sil supplementation decreased the MR% in those consuming AFB1-contaminated diets, with RLP% ranging from 33.3% to 100%. Antibiotic treatment resulted in high MR%, whereas SDZ and TMP did not. While SD&TMP or Cip treatment in combination with dietary Sil achieved low MR% of 50% to 62.5% and 50% to 71.43%, respectively, and low RI% of S. agalactiae of 30% to 50% and 20% to 50%, respectively.

**Table 3:** 10% of LD_50_ of *S. agalactiae.*Mortality rate (MR), relative protection level (RLP), and pathogen re-isolation (RI). (*n* = 10).

Groups	Antibiotic free			SDZ & TMP			Ciprofloxacin		
	MR (%)	RLP (%)	RI (%)	MR (%)	RLP (%)	RI (%)	MR (%)	RLP (%)	RI (%)
Control (-ve)	10	-	-	-	-	-	-	-	-
Control (+ ve) AFB0	20	-	100	10	-	0	0	-	0
AFB50	30	-	60	40	-	70	40	-	60
AFB100	60	-	100	70	-	100	70	-	100
AFB150	80	-	100	80	-	100	70	-	100
AFB0 + Sil	20	0	40	0	100	30	0	100	20
AFB50 + Sil	20	33.3	50	20	50	40	20	50	30
AFB100 + Sil	40	33.3	80	30	57.14	50	20	71.43	40
AFB150 + Sil	40	50	80	30	62.5	50	20	71.43	50

Different letters in the same column indicate significant differences between values at *P* ≤ 0.05. AFB0 (control, uncontaminated diet), AFB50 (50 ppb AFB_1_), AFB100 (100 ppb AFB_1_), AFB150 (150 ppb AFB_1_), AFB0 + Sil (0 ppb AFB_1_+100 mg silymarin), AFB50 + Sil (50 ppb AFB_1_+100 mg silymarin), AFB100 + Sil (100ppb AFB1 + 100 mg silymarin), and AFB150 + Sil (150 ppb AFB_1_+100 mg silymarin).

## Discussion

Accumulation of AFB_1_ in fish tissues depends on many factors, such as exposure level and rate; absorption surface and rate; metabolism and excretion rate^7^. In this study, AFB_1_ gradually accumulated in the hepatic tissues of the exposed Nile tilapia at levels of 0.34, 0.54, and 0.82 ppb after receiving contaminated diets at concentrations of 64.2, 122.4, and 171.8 ppb, respectively, for 84 days, meanwhile the muscular tissues showed residue traces. In accordance, channel catfish were orally administered a single dose of AFB at 250 ppb b.w., which corresponds to a diet concentration of 8000 ppb. Despite the high dose being administered the residues in the muscular tissue were still below the determination limit (< 5 ppb) after 24 h^7^, lower than the permissible limit (20 ppb) approved by the FDA^43^. Similarly, Deng et al.^44^ found that Nile tilapia fed AFB_1_-contaminated feed at concentrations of 19 ppb, 85 ppb, 245 ppb, 638 ppb, 793 ppb and 1641 ppb for 20 weeks, after 15 weeks, aflatoxins residues were noticeable in the hepatic tissue in those received 85 ppb and higher concentrations, they resulted in residue levels ranged between 10.2 ppb and 24.0 ppb, while after 20 weeks, residues in the hepatic tissues ranged from 30.4 ppb to 47.4 ppb. Also, Hussain et al.^45^, reported that walleye fish (*Sander vitreus vitreus*) fed AFB-contaminated feed at levels of 50 ppb and 100 ppb for 30 days, their muscular tissues had AFB_1_, AFB_2_, AFG_1_, and AFG_2_ at concentrations of 5, 10, 15, and 20 ppb, respectively. These differences are attributed to the sensitivity of fish species to AFB1, so the study concluded that tilapia species are less susceptible than other fish species^46^.

In this experiment, dietary AFB_1_ induced oxidative stress and immunosuppression status in experimental fish, the expressions of *GPx*,* SOD*,* IL-1β*, and *TNF-α* genes were linearly declined as the level of AFB_1_ increased, CAT expression was increased in AFB50 group and was declined with higher the AFB_1_ levels. Similarly, exposure to dietary AFB_1_ could reduce the expression of antioxidant-related genes *CAT*,* SOD*, and *GPx* in the hepatic tissues of turbot^47^, Nile tilapia, roha, sea bass, and grass carp^48^. Some researchers found a rise in the expressions of previous genes, healthy hybrid grouper received dietary AFB_1_ at different levels of 0, 7, 30, 111, 445, and 2230 ppb, exhibited a significant upgrade of inflammatory responses (*TNF-α* and *INF-α*) and high ROS biomarkers (*keap1*), reducing antioxidant capacity (*nrf-2* and *gpx*) dose-dependent in the hepatic tissue^49,50^. Also, gene expression of pro-inflammatory factors *NF-kb*,* TNF-a*,* IL-1β*,* IL-6*,* IL-8*, and *IFN-γ;* anti-inflammatory factors *IL-10*,* Tgf-β*, and antioxidant regulatory factors *nrf2/keap1* were up-regulated in the hepatic and intestinal tissues of largemouth bass (Micropterus salmoides) challenged with mycotoxin for eight weeks^51^. While the expression of *IgM-2* and *IGF1* genes declined in hepatic tissues in a dose-dependent manner in response to dietary AFB1 in the experimental Nile tilapia, mycotoxins induce immunotoxicity, which adversely impacts the production of immune response mediators and impairs immunocompetent cells, leading to disruption of the innate and adaptive immunity^52^. Innate immunity of the experimental Nile tilapia (SAB, OBA, PA, PI, and LYZ) was negatively impacted dose-dependently with AFB1. Similarly, Nile tilapia fed AFB1-contaminated diets (1.5 and 3.0 ppm) for 10 weeks exhibited a significant decline in the hepatosomatic index and macrophage extracellular superoxide anion production^5^. Similar findings were obtained in murine species^53,54^. Experimental Nile tilapia fed AFB1-contaminated diets showed high MR%. These findings were attributed to AFB-induced declines in free radicals scavengers and free radical were deployed to kill invading pathogen^55^, it is well known that AFB drastically impairs the fish immune system, rendering fish more sensitive to environmental stress and more vulnerable to microbial infections^8,56^.

From the obtained results, dietary Sil supplementation could partially restore the immune-antioxidant activities enhancing the expression *IL-1β*,* TNF-α*, and *IL-10* genes. Similarly, the levels of *GPx* and *CAT* increased in Nile tilapia liver after 6 weeks of dietary-Sil then restored the baseline levels^57^, also SOD and CAT activity were upregulated compared to the control fish^58^. Similar results obtained in previous studies, Sil supplementation has immune-antioxidant properties that could protect animal cells by scavenging the propagated ROS and suppressing the inflammatory process via increasing the expression of *TNF-α*, nitric oxide synthase *(iNOS)*, interleukins *(IL-1β*,* IL-6*,* and IL-10)*, and messenger RNA in a dose-dependent manner^59–61^. Moreover, administration of Sil at concentrations up to 10 mg/kg can suppress leukocyte function, whereas higher doses (50–250 mg/kg) stimulate inflammatory processes^62^.

Experimental Nile tilapia fed AFB_1_-contaminated diets were challenged with a sub-lethal dose of *S. agalactiae* (10% of LD_50_) in high MR%, whereas Sil acted synergistically with SDZ & TMP and Cip to decrease mortality. In accordance, aflatoxicosis (1.177 ppm) accompanied by *A. hydrophila* infection acted synergistically and resulted in high MR%^63^, whereas dietary-Sil could decrease MR% compared to the control fish provided RLP of 25%^61^. Furthermore, about 28% of Nile tilapia survived *Streptococcosis* after receiving dietary-Sil while all the control fish died after 192 h^57^. Furthermore, Sil-supplementation could mitigate the intravascular impacts of *A. hydrophila* infection; however, late Sil supplementation could not ameliorate cellular degeneration caused by bacterial infection^64^. In the same line, herbal additives (Rutin) could improve oxytetracycline efficacy in silver catfish by exerting hepatoprotective effects, reducing oxidative stress and apoptosis^65^. Compared with the control group, Nile tilapia fed dietary-Sil for 8 weeks had higher SR (95%) after 2 weeks^61^. Since AFB_1_ inhibits hepatic cytochrome P450 monooxygenases, altering the tissue residues of drugs that are predominantly metabolized in the hepatic tissues^66,67^, the active metabolites of ciprofloxacin are metabolized to oxociprofloxacin and desethyleneciprofloxacin, which do not possess the antibacterial effects^68^.

## Conclusion

Sil-supplementation decreases the residues of AFB1 in the hepatic tissues of the Nile tilapia, and ameliorates the adverse impacts of AFB1 on antioxidant-immune status by raising the expression of GPx, SOD, CAT, IL-1β, TNF-α, IL-10, IgM-2, and IGF genes in the hepatic tissue as well as innate immunity. Finally, Ciprofloxacin and sulfadiazine & trimethoprim could increase RLP in fish exposed to AFB1-contaminated diets for 84 days, supported by the Sil supplementation.

## Data Availability

Data are available on request from the corresponding author.
